# Spent hemp biomass in broiler diets: effects on performance, meat quality, expression of genes in internal adipose tissue, and corticosterone in feathers

**DOI:** 10.1016/j.psj.2026.107692

**Published:** 2026-08-28

**Authors:** Nathan B. Parker, James Hermes, Agung Irawan, Carolyn Pearce, Dresen Ferschweiler, Serkan Ates, Massimo Bionaz

**Affiliations:** a Department of Animal and Rangeland Sciences, Oregon State University, Corvallis, Oregon, 97331, USA; b Universitas Sebelas Maret, Surakarta, Central Java, 57126, Indonesia

**Keywords:** Spent hemp biomass, Broilers, Performance, Meat quality, Gene expression

## Abstract

Prior use of spent hemp biomass (**SHB**), a byproduct of cannabidiol extraction, has shown promise as a low-cost livestock feed in ruminant diets. However, due to concerns over residual cannabinoids in carcass tissues, it has not been approved by the FDA-CVM for livestock use. Moreover, research on SHB effects in monogastrics remains scarce. The objective of this study was to examine varying levels of dietary SHB inclusion in broiler diets and their effects on performance, meat quality, expression of genes in adipose tissue, and stress. Two hundred (n=200) 5-day-old Cobb-500 chicks were randomly assigned to corn-soybean-based diets containing 0%, 5%, 10%, or 20% SHB for a 42-day feeding trial (5 pens/group). Diets were *isocaloric, isonitrogenous,* and balanced for fiber. At the end of the experiment, birds were euthanized and sexed, with two males and two females selected from each pen (n=80) for subsequent sampling. Feed intake was not adversely affected by SHB supplementation; however, inclusion at ≥10% negatively impacted feed conversion rate and body weight, while pasty vent was higher in all SHB treatments than in the control. Dietary SHB at ≥10% level decreased growth and breast weight. SHB at 20% level increased breast yellowness (b*), drip loss (vs. lower SHB doses), and shear force (i.e., lower tenderness). Feeding 20% SHB decreased triglycerides, total fatty acids, and the proportion of unsaturated fatty acids in muscle (vs. lower SHB doses), while increasing the proportion of cholesterogenic and bacteria-derived fatty acids (vs. lower SHB doses), suggesting SHB affects lipid synthesis and utilization. This was confirmed by the increase in transcription of genes involved in fat catabolism, *ACAA1* and *ACOX,* in the abdominal adipose tissue, specifically for males. Birds receiving 20% SHB also had higher corticosterone in the feathers, indicating possibly higher chronic stress. Overall, dietary SHB inclusion at levels ≥10% negatively affected broiler performance, meat quality, lipid metabolism, and corticosterone in feathers (as a chronic stress indicator), whereas inclusion at 5% did not produce adverse effects. These results indicate that low levels of SHB may be incorporated into broiler diets without compromising bird health or productivity, although higher inclusion rates appear detrimental.

## Introduction

Since its removal from the Comprehensive Drug Abuse Prevention and Control Act with the passage of the 2014 and 2018 Farm Bills, hemp (*Cannabis sativa* L.) cultivation and use in the USA have experienced rapid growth and public interest (Irawan et al., 2025). Well-known for its versatility as an agricultural commodity, hemp is utilized across many industries and sectors, including construction materials, paper, textiles, and biofuels (Ahmed et al., 2022), in addition to medicinal and nutraceutical applications in humans (Visković et al., 2023). The utility of hemp has made it an economically significant crop in both international and domestic markets. In 2022, the USA industrial hemp production was valued at $238 million (USDA NASS, 2022).

In recent years, hemp has also demonstrated viability in turning low-value byproducts into wholesome feed sources for livestock, improving animal health, and optimizing food quality attributes (Irawan et al., 2025). Of particular interest is spent hemp biomass (**SHB**), a byproduct of the cannabinoid extraction process. This by-product primarily consists of leaf and flower material from the inflorescence of the plant. Currently, SHB has little economic value, yet can be used as a feed ingredient for livestock. Recent research has demonstrated that SHB possesses a nutritional profile comparable to alfalfa and can be utilized in ruminant diets without detrimental effects on animal health, performance, or food quality (Parker et al., 2022; Irawan et al., 2024; Irawan et al., 2025).

Regardless of the benefits exhibited by hemp when used in animal diets, regular utilization of hemp in livestock diets in the USA has yet to receive approval from the Food and Drug Administration, Center for Veterinary Medicine. This is due to concerns over residual levels of cannabinoids, especially cannabidiol (**CBD**) and the psychoactive tetrahydrocannabinol, resulting in hemp’s classification as a drug and negating its use in livestock feed (FDA, 2018). Moreover, any food products sourced from livestock fed hemp are considered adulterated by the United States Department of Agriculture, further limiting their implementation in dietary rations (USDA, 2023).

Little is known regarding the SHB effect on monogastric species. Previous studies evaluating hempseed cake in the diet of layer hens (Kasula et al., 2021a) reported no adverse health effects on bird growth and performance parameters but enhanced the nutritional status of eggs and meat via lipid profile alterations. Additionally, Kasula et al. (2021b) did not detect any cannabinoid residues in eggs from hens consuming hempseed cake.

The nutritional composition of SHB differs from hempseed, especially with respect to protein, fat, and fiber content (Irawan et al., 2025). Thus, it is difficult to draw comparisons from trials utilizing hempseed and other hemp byproducts to those of SHB, especially since there is a paucity of research evaluating SHB in monogastric diets. Based on the fact that monogastrics digest and deposit dietary constituents differently from ruminants (Wood et al., 2008), it is essential to investigate the impact of SHB on monogastric animals with respect to animal health, performance, and product quality. Prior studies have investigated the use of SHB in broilers (Sopian et al., 2024; Sopian et al., 2025), with an overall positive impact on the animals. However, the level used in the diet was low (up to 2%) and used as a nutraceutical supplement, not as a main dietary source. Thus, it remains to be assessed whether the use of high levels of SHB in the diet of broiler chickens is feasible. Ultimately, this information could be used in policy-making decisions regarding the legalization of hemp as livestock and poultry feed, in concert with increasing the economic viability of SHB as a commodity. To that end, the objective of this study was to evaluate the effect of varying levels of SHB inclusion in broiler diets on bird performance and meat quality, evaluating the effect on organ weights, adipose gene expression, stress (by measuring corticosterone in feathers), and sex-related responses.

## Materials and methods

All procedures were approved by the Oregon State University (OSU) Animal Care and Use Committee (ACUP# 0192) before the commencement of the experiment. The study was carried out at the Poultry Center on the OSU campus (Corvallis, OR, USA).

### Experimental design, animals, and diets

A diagrammatic illustration of the experimental design is available in Figure 1. In the study, Cobb-500 Cornish-cross broiler chicks (n=200; 5 d old) were obtained from a local hatchery and randomly assigned to 20 pens (10 birds/pen; 5 pens/treatment). Chicks were individually tagged using a metal leg tag with an individual number and weighed immediately after arrival. The chicks had an average starting body weight (**BW**) of 48.3±1.8 g. After weighing, chicks were randomly assigned to one of four corn-soybean meal-based poultry rations containing 0% (**CON**), 5% (**SHB5**), 10% (**SHB10**), or 20% SHB (**SHB20**). The composition of the diets is available in Table 1. All ingredients, except SHB, were purchased from CHS Nutrition, Harrisburg, Oregon. Chicks were housed in an environmentally controlled poultry house with a lighting program of 23L:1D and were not vaccinated. Pens (1.5 × 2.0 m) contained wood shavings and free access to water.

**Figure 1:**
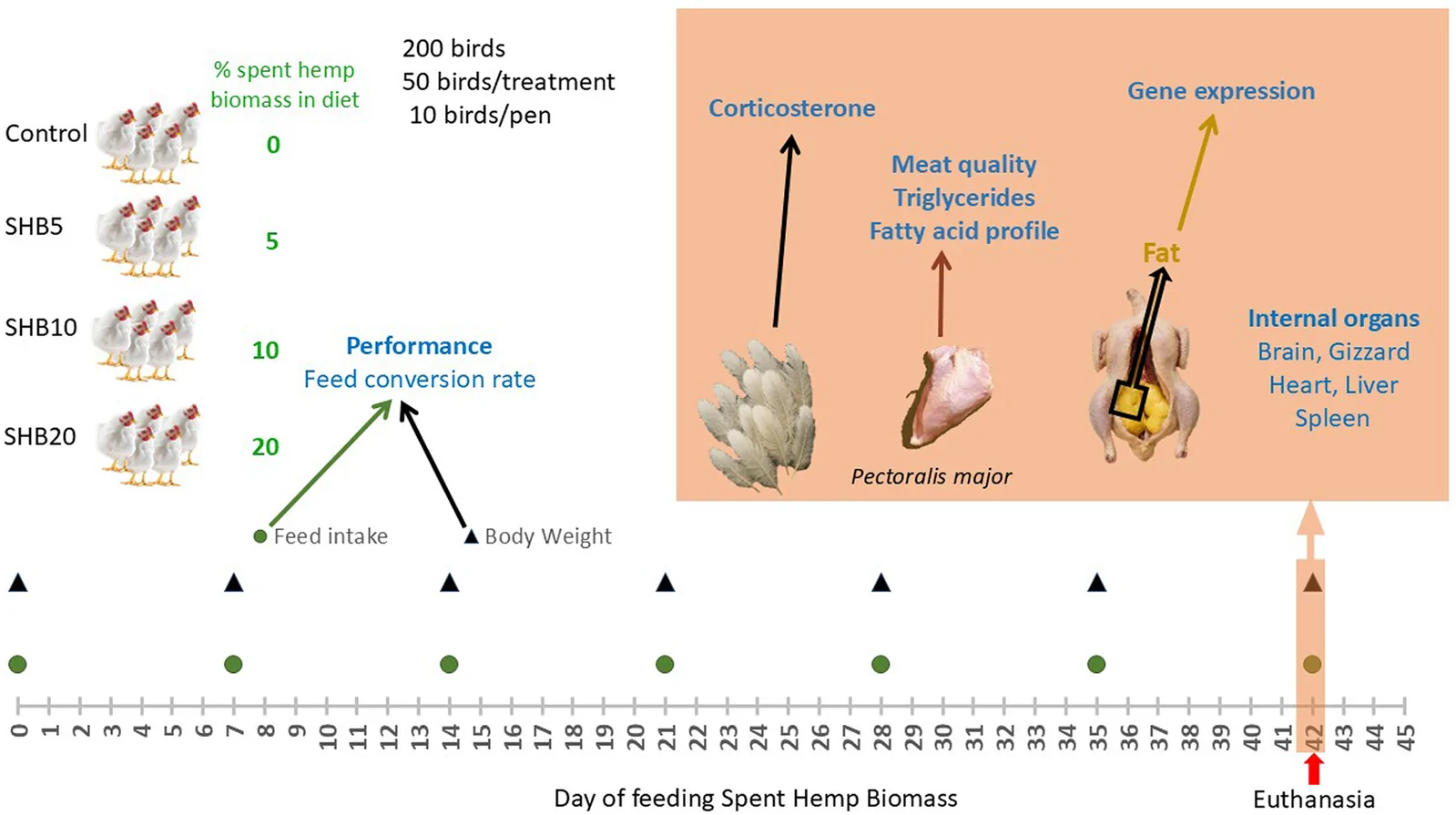
Experimental design of broilers that were fed diets containing 0, 5, 10, or 20% spent hemp biomass (SHB).

**Table 1:** Formulation and chemical composition of experimental broiler diets (DM basis).

**Items**		**Control**	**5%**	**10%**	**20%**
Ingredient*^1^					
Corn, grain	%	42.0	41.5	40.5	37.0
Soybean meal -46%	%	37.8	36.0	34.0	31.0
Limestone	%	2.0	2.0	2.0	2.0
Fertrell Poultry Nutri Balancer	%	3.0	3.0	3.0	3.0
Spent hemp biomass	%	0.0	5.0	10.0	20.0
Rice hulls	%	7.2	5.5	4.0	1.0
Soybean oil	%	8.0	7.0	6.5	6.0
***Chemical composition***					
DM	%	88.7	88.7	88.2	88.6
CP	% DM	22.8	24.6	23.8	23.6
Soluble protein	% DM	4.7	6.6	4.8	4.7
ADF	% DM	4.1	7.8	8.4	8.1
NDF	% DM	11.9	13.3	13.3	13.3
Lignin	% DM	2.23	2.06	2.21	2.09
Crude fat	% DM	10.1	9.39	9.08	9.51
Ash	% DM	8.38	9.37	8.81	9.27
Ca	% DM	1.43	1.56	1.67	2.06
P	% DM	0.76	0.80	0.83	0.86
Ca/P		1.88	1.95	2.01	2.40
Mg	% DM	0.25	0.28	0.31	0.37
K	% DM	1.17	1.28	1.33	1.47
Na	% DM	0.19	0.20	0.18	0.20
Fe	ppm	292	303	320	345
Mn	ppm	101	107	126	156
Zn	ppm	92	120	123	131
Cu	ppm	48	18	19	41
TDN	% DM	86.8	84.5	84.4	84.6
ME	Mcal/kg	3.42	3.31	3.31	3.33

⁎ All ingredients, except for the spent hemp biomass were purchased from CHS Nutrition, Harrisburg, Oregon

SHB used in the study was provided by the Columbia Hemp Trading Company (Corvallis, OR). Composition of the SHB, including cannabinoid profile, mycotoxin levels, and other parameters, has been previously reported (Parker et al., 2022). Pelletized hemp was ground (< 1mm) using a hammer mill grinder (Southern Equipment Company, Goodlettsville, TN), then mixed using a commercial mixer with the rest of the ingredients at either 5, 10, or 20% by weight, respectively.

Chicks were fed *ad libitum,* and all dietary treatments were isocaloric, isonitrogenous, and balanced for fiber using rice hulls. Feed intake, body weight, and pasty vent scores were recorded every 7 d throughout the trial. Pasty vent was scored as outlined in Figure S1 in the Supplementary File. The weekly feed conversion rate for each pen (**FCR;** g feed/g weight gain) was calculated correcting for mortality using the following formula:

$$FCR=\;\frac{Total\;weekly\;feed\;consumption\left(g\right)}{Live\;weight\;gain\;\left(g\right)+Estimated\;weight\;gain\;of\;death\;animals\;\left(g\right)}$$

The average weight of the animals in the same pen was used to estimate the weight gain of the dead animal(s).

The intake of CBD was estimated using the amount of SHB in the diet, the measured CBD in the SHB (Parker et al., 2022), the weekly feed intake, the number of birds in each pen, and the weekly BW.

### Feed samples and analyses

Feed samples from each diet were collected after each mixing (3 batches total). Samples were shipped to a commercial laboratory (Cumberland Valley Analytical Services, Waynesboro, PA, USA) for wet lab proximate and amino acid composition analyses. Feed was also analyzed for fatty acid (**FA**) composition as described below.

### Sample collection

On days 42, 43, and 44 of the trial, birds were weighed, and an equal number of birds from each dietary treatment was transported to the Oldfield Animal Teaching Facility, where they were euthanized via CO_2_ stunning and exsanguinated by decapitation after hanging the animals. After exsanguination, the carcass was eviscerated, and internal organs, brain, and breast muscles (*pectoralis major*) were collected and weighed. Birds were also sexed post-euthanasia, and the 2 males and the 2 females with the BW closer to the average for each sex and pen were selected (i.e., the birds with the extreme BW were removed) for subsequent analysis.

### Meat quality assessment

Fresh (unfrozen) breast samples from 2 males and 2 females per pen (4 per pen; 20 per treatment) were deboned, trimmed of excess fat, and then transported to the Clark Meat Science Center at Oregon State University, where they were used for meat quality analyses. Breast samples from the right half of the carcass were weighed after slaughter and then used to determine pH, instrumental color, and drip loss. After analyses, right breast samples were vacuum-sealed using a single-chamber machine (Model SC-680, Promarks Inc. Ontario, CA) in 20.3 × 30.5 cm pouches (Clarity™ 3-Mil nylon, Bunzl Processor Division, Kansas City, MO) and stored at -20°C until further examination of fatty acid composition and total triglycerides (**TG**). Left breast samples from each replicate were excised post-mortem and refrigerated in a walk-in cooler at 4°C±1. Samples were weighed at ∼12 h post-mortem, then vacuum-sealed in 20.3 × 30.5 cm pouches and frozen at -20°C until evaluation for cook loss and tenderness.

### Instrumental color evaluation

Before color measurement, breasts were blotted dry with a paper towel, then packaged in 21 x 14.6 × 1.9 cm black polystyrene trays (CKF Inc., Langley, B.C., Canada) with a soaker pad (Dri-loc AC-25, Sealed Air Cryovac, Duncan, SC) in each tray. Breasts were overwrapped using a PVC overwrap machine (Model 625, Heat Sealing Mfg. Co., Cleveland, OH) with clear, stretch O_2_ permeable film (RMF 61-HY). Instrumental color readings were carried out in a commercial retail cooler, 4°C±1 at ∼24 h post-mortem under refrigerated storage using a HunterLab MiniScan EZ portable spectrophotometer (Model 45/0 LAV, Hunter Associates Laboratory, Inc. Reston, VA) with a 1.54 cm aperture, calibrated with black and white standards. Three color value readings were measured on each breast at three locations: two at the rostral part (thick part of fillet) and one in the middle. The Commission Internationale de l’Eclairage L*, a*, and b* tristimulus color space values were calculated (CIE, 1978), and values were averaged for each sample.

### Drip loss

Immediately after instrumental color readings were performed, breasts were evaluated for 24 h drip loss. Breast samples were removed from polystyrene tray packaging and blotted dry with a paper towel prior to weighing. Weights were recorded using a digital scale (Model SP6001, OHAUS Corporation, Parsippany, NJ) at 0, 12, and 24 h, and drip loss percentage was calculated using the following equation:

Drip loss (%) = [Initial weight of breast (g) – final weight of breast (g)] /Initial weight of breast (g) x 100

Breasts were stored in Ziplock bags in a refrigerated cooler 4°C±1 during drip loss analysis. Samples were blotted dry with paper towels, and new Ziplock bags were used between measurements (after 0 and 12 h weight recordings) to avoid erroneous weights from water contamination.

### pH evaluation

Muscle pH was measured ∼48 h post-mortem following color and drip loss analyses in a commercial retail cooler at 4°C±1. A meat pH/temperature meter (Model HI99163, Hanna Instruments, Smithfield, Rhode Island) equipped with a knife attachment was used. The meter was calibrated in 7.0 and 4.0 pH buffers prior to measurements. The probe with knife attachment was inserted ∼1.5 cm into the rostral part of the breast, and two readings were taken. The probe was rinsed between each sample with distilled water. Readings were averaged to yield the mean value of each sample.

### Cook loss

Prior to cook loss evaluation, samples were thawed for 24 h in a commercial retail cooler at 4°C±1. Breasts were blotted dry with a paper towel and weighed prior to cooking using a digital scale. Breasts were cooked using a conventional oven set at 176°C±5 as previously described (Khan et al., 2021). One breast from each treatment was placed on an elevated rack on a baking tray with foil lining during each cook cycle (4 breasts per cycle). Oven temperature was monitored via a thermocouple (copper constantan type T). The temperature of the breasts was monitored using a thermocouple inserted into the geometric center of the fillet using a needle, then cooked until an internal temperature of 76°C±1 was reached. Temperature was monitored and recorded using PicoLog 6.1.10 software (Pico Technology, Tyler, TX). Samples were removed from the oven and allowed to cool for 30 min at room temperature (21°C±1). After cooling, samples were blotted dry with a paper towel, and the final cooked weight was recorded. Cook loss percentage was calculated using the following equation:

Cook loss (%) = [Initial weight before cooking (g) − final weight after cooking (g)]/ Initial weight before cooking (g) x 100

After weighing, samples were wrapped in foil and stored at 4°C±1 for 24 h prior to texture analysis.

### Texture analysis

Breast samples were evaluated for texture (tenderness) using the Warner-Bratzler shear force method. Three 1.27 cm core samples were taken from each cooked breast at the rostral and middle sections of the fillets, in the longitudinal orientation of the muscle fiber. Samples were sheared in the center of each core using a Shimadzu EZ-X Series Tabletop Testing Machine (Shimadzu Corporation, Japan) with a load cell size of 500 N. Travel distance was set at 30 mm and travel speed at 4 mm/s. Sample values were recorded and analyzed using Trapezium X version 1.4.0 software (Shimadzu Scientific Instruments, Columbia, MD) and averaged for each breast. Shear force values were reported in Newtons (**N**).

### Triglycerides and fatty acid analysis

The quantification of TG in the breast was performed in duplicate using a commercial kit (cat#10010303, Cayman Chemical, Ann Arbor, Michigan, USA) following the manufacturer’s instructions; the intra-assay had a coefficient of variation of 5.7%. The FA profiles of the breast collected after euthanasia, in addition to feed samples from SHB and dietary treatments, were measured and analyzed using the method described by Folch et al. (1957), with modifications. Briefly, samples (approx. 1 g) were weighed and extracted using 3 mL of 2:1 chloroform: methanol and homogenized in a tube using a tissue tearer (Model 985370, Biospec Products, Inc., Bartlesville, OK). After homogenization, samples were held overnight at 3°C±1, mixed with 2 mL of 0.88% NaCl solution and 100 μL of 30 mg/mL nonadecanoic acid (C19:0) as the internal standard (cat# 11909, Sigma-Aldrich, St. Louis, MO), and then centrifuged at 2.850 × g for 10 minutes. The bottom layer containing the lipid extract was transferred to a glass vial with a screw cap and dried under a gentle flow of nitrogen gas. After drying, 2 mL of 3 M hydrochloric acid solution in Methanol (cat#07607, MilliporeSigma, USA) was added to the vial, then heated at 90°C for 60 min with periodic shaking to produce the FA methyl esters. After cooling in a water bath, 1 mL of hexane and 1 mL of distilled water were added to the vial, then shaken for 2 min. Upon separation, the upper phase hexane layer was transferred into a 2 mL autosampler GC vial. The FA analysis was performed using a 100 m × 0.25 mm × 0.2 µm Fused Silica capillary column (Cat #23362-U, SP-2560 Supelco, MilliporeSigma) in an Agilent 6890 gas chromatograph (Agilent Technologies Inc., Palo Alto, CA) equipped with an autosampler and flame ionization detector. Identification of peaks was achieved via the 37 Component FAME Mix certified reference (Cat# CRM47885, MilliporeSigma, USA), and each FA was calculated as the relative % area of the annotated peaks and as µg/g of tissue, by correcting each peak for the known amount of internal standard after correction for the FID response of each FA. The temperature program of the GC was set using the previously reported method (Irawan et al., 2024).

### RTqPCR

A sample of the abdominal adipose tissue was collected at slaughter in a 1.5 mL cryovial and immediately flash-frozen in liquid N. Once in the laboratory, the samples were preserved at -80°C until analysis. The RNA was isolated using the RNeasy Lipid Tissue Mini Kit (cat# 74804, QIAGEN, USA) following the manufacturer’s instructions. RNA purity and integrity were assessed using a NanoDrop One spectrophotometer (ThermoFisher, USA) and Bioanalyzer 2100 Agilent, USA), respectively. The OD280/OD260 was 2.05±0.03, and the RNA Integrity Number was 6.9±0.9, ranging from 5.4 to 8.9. Primer design, cDNA synthesis, and PCR were performed exactly as previously described (Rosa et al., 2017). The final RTqPCR data were obtained using LinRegPCR (Ruijter et al., 2009). Four reference genes were tested (*GAPDH, MRPL39, MRPS39*, and *UXT*) using geNorm (Vandesompele et al., 2002). A reliable normalization was obtained by using the geometric mean of *GAPDH, MRPS39*, and *UXT* with a V-value of 0.239. The primers of the reference genes, plus 9 target genes coding for proteins involved in FA activation and transport (*ACSL1* and *FABP4*), lipogenesis (*LPL, DGAT2*, and *SREBF1*), lipid oxidation (*ACAA1, PPARA*, and *ACOX1*), and glucose uptake (*SLC2A4*), are available in Table S1 in the Supplementary File.

### Corticosterone in feathers

Feathers from the wings were plucked immediately after euthanasia, placed in a labelled paper envelope, and preserved at -20°C until analysis. Corticosterone in feathers was extracted following previous methods by Freeman and Newman, (2018) and Krueger et al. (2025) with modifications. Briefly, approximately 50 mg of feathers without any visible debris, including blood, were cut with scissors, precisely weighed, and placed in a 30 mL glass tube containing 10 mL of methanol. The feathers were processed in methanol using a tissue tearer (Model 985370, Biospec Products, Inc., Bartlesville, OK). When no large particles were observed, the tube was sonicated for 30 minutes and incubated at 37°C overnight in a shaking water bath. Samples were then preserved at -20°C until further processing. Samples at room temperature were centrifuged at 4000 × g for 10 minutes. The supernatant was collected in a 5 mL vial and evaporated at 45°C. A commercial kit (cat# K014-H1, Arbor Assay, USA) was used to quantify the corticosterone after evaporation following the manufacturer’s instructions. The intra-assay variation was 9.7%.

### Statistical analyses

Data with a time component (i.e., feed intake, BW, FCR, and fecal score) were analyzed using a Proc GLIMMIX with an AR (1) covariance structure, with birds nested in pen (n=5 pens per treatment, 4 birds/pen) as a random effect and treatment, sex, time, and their interactions as fixed effects. We used PROC GLM for all other parameters, with treatment, sex, and their interaction as fixed effects. Before statistical analysis, outliers were identified using PROC REG in SAS (v9.4), and data with a studentized t ≥ 3 were removed. Significance was set at *P* ≤ 0.05, with values between >0.05 and 0.10 considered to have a tendency. PROC FREQ in SAS was used to analyze the frequency of pasty vent, and PROC GLM was used to analyze the linear and quadratic effects of the SHB dose.

## Results

### Nutritional composition of diets

Results of the chemical analysis of the diets are available in Table 1. Diets between groups were generally balanced; however, some differences were observed, with, in birds receiving 5, 10, and 20% SHB compared to CTR, higher CP (+7.9%, +4.4%, and +3.5%, -respectively), NDF (+11.8% in all SHB groups) and ash (+11.9%, +5.1%, and +10.6%, respectively) but lower lignin (-7.6%, -0.9%, and -6.3%, respectively), fat (-7.0%, -10.1%, -5.8%, respectively), and metabolizable energy (-3.2%, -3.2%, and -2.6%, respectively). Additionally, all minerals except Cu and Na increased linearly with higher dietary SHB, including the Ca/P ratio, which reached 2.4 in SHB20, which was +27.7% higher than CTR. Amino acid and FA profiling were well-balanced among diets (Tables S2 and S3 in the Supplementary File).

### Performance

Mortality was <4% in all groups, with lower mortality in SHB5 (2%) than in the other groups (3.8%) (Figure S1 in the Supplementary File). Feed intake throughout the trial was not affected by the inclusion of SHB in the diet (Figure 2). However, SHB at 10% and 20% inclusion negatively affected animal growth, as indicated by BW and FCR (Figure 2). The latter was significantly larger in birds receiving 10% and 20% vs. 5% and 0% SHB in the diet during the first 3 weeks of the dietary treatment. The birds consumed, on average during the whole experiment (mean±SD), 29±8, 68±30, and 172±72 mg CBD/kg BW for 5%, 10%, and 20% SHB groups, respectively, with a higher mg CBD/kg BW intake during the first 3 weeks of the experiment (Figure 2). An overall sex effect was observed for BW, as the weight of the males was larger than the weight of the females, with no interaction of Sex × SHB (Figure S3 in Supplementary File).

**Figure 2:**
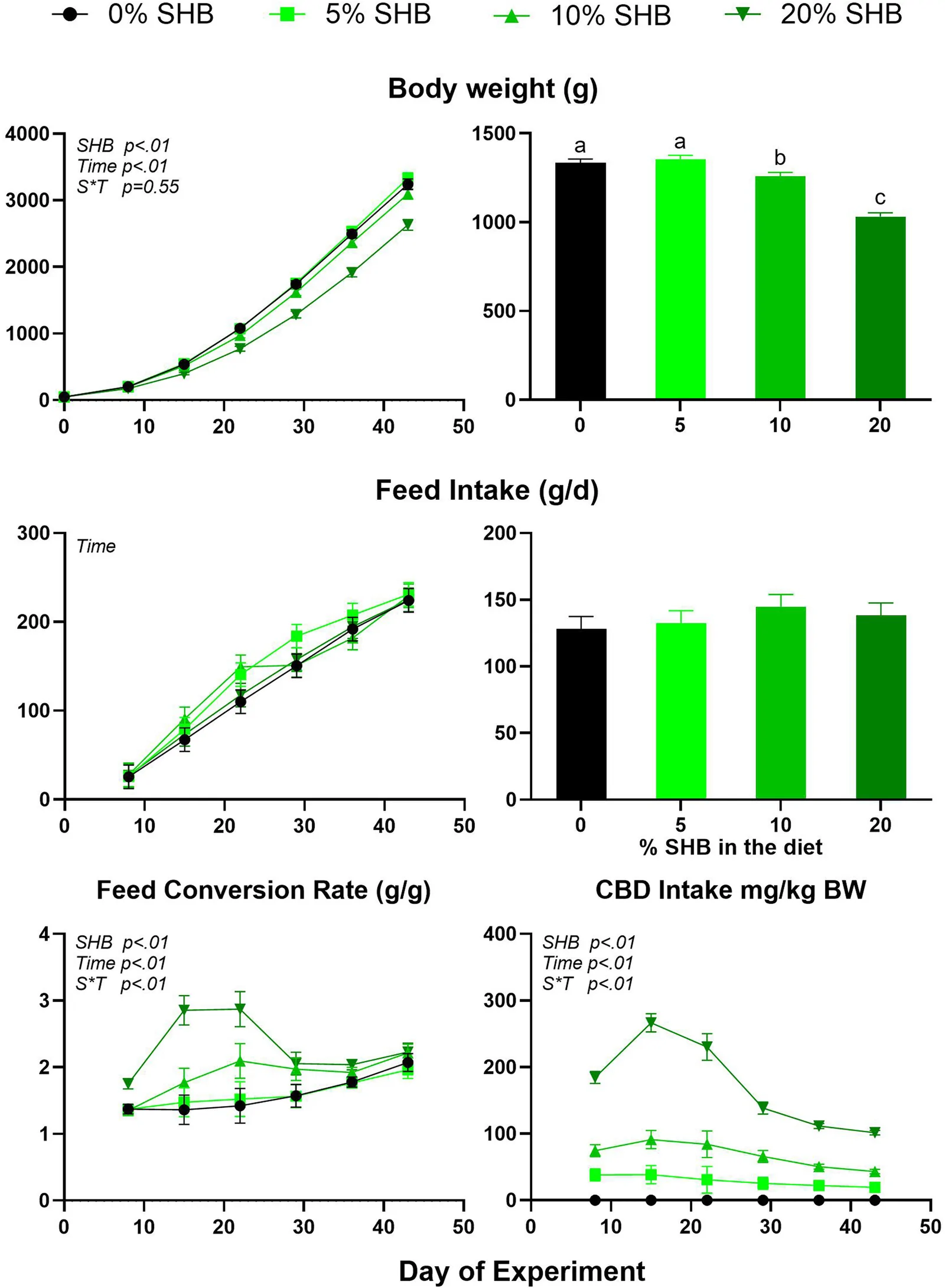
Body weight, feed intake, feed conversion rate, and intake of cannabidiol (CBD) in broiler chickens receiving 0, 5, 10, or 20% dietary spent hemp biomass (SHB). For all parameters, the changes through time during the experiment (left graph for body weight and feed intake) are reported. For body weight and feed intake, the overall mean of those parameters over the whole experimental period is reported (right graphs).

### Pasty vent score

The addition of SHB in the diets increased the frequency and amount of pasty vent in birds, with all SHB groups experiencing a similar increase in pasty vent compared to CON (Figure 3). However, no overall Sex or SHB × Sex effects were noted for pasty vent score (Figure S4 in Supplementary File).

**Figure 3:**
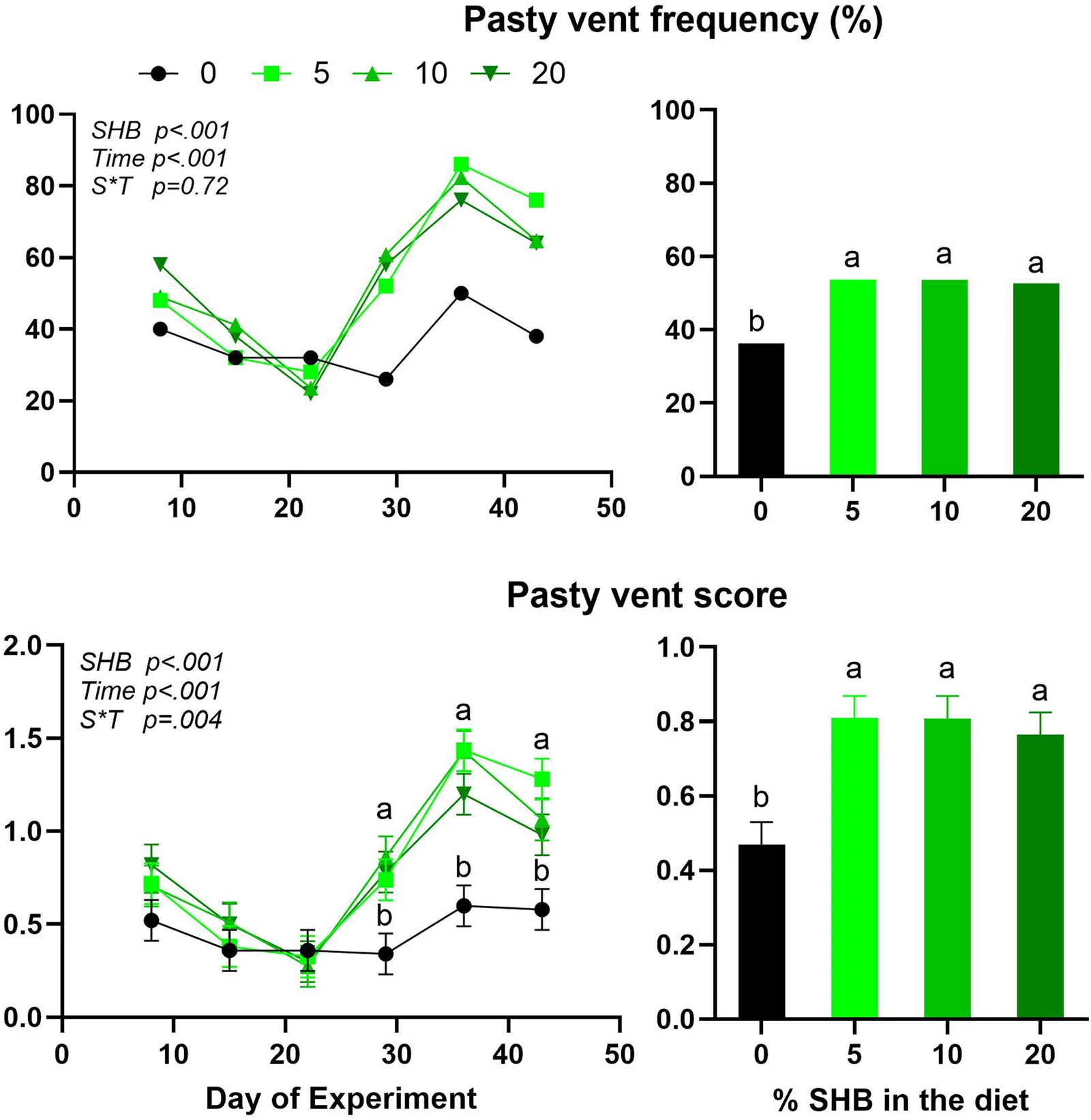
Pasty vent frequency and score in broiler chickens receiving 0, 5, 10, or 20% dietary spent hemp biomass (SHB). Letters denote statistical differences (p<0.05) between groups at the same time point when an interaction SHB × Time was significant (p<0.05).

### Meat quality

Feeding 20% SHB resulted in lower weight and triglyceride content of the breast (Figure 4). The breast was smaller for females than males, while the triglyceride content was affected by feeding SHB differently between males and females, with females having a higher content of triglyceride when fed 10% SHB in the diet compared to males (Figure S5 in Supplementary File).

**Figure 4:**
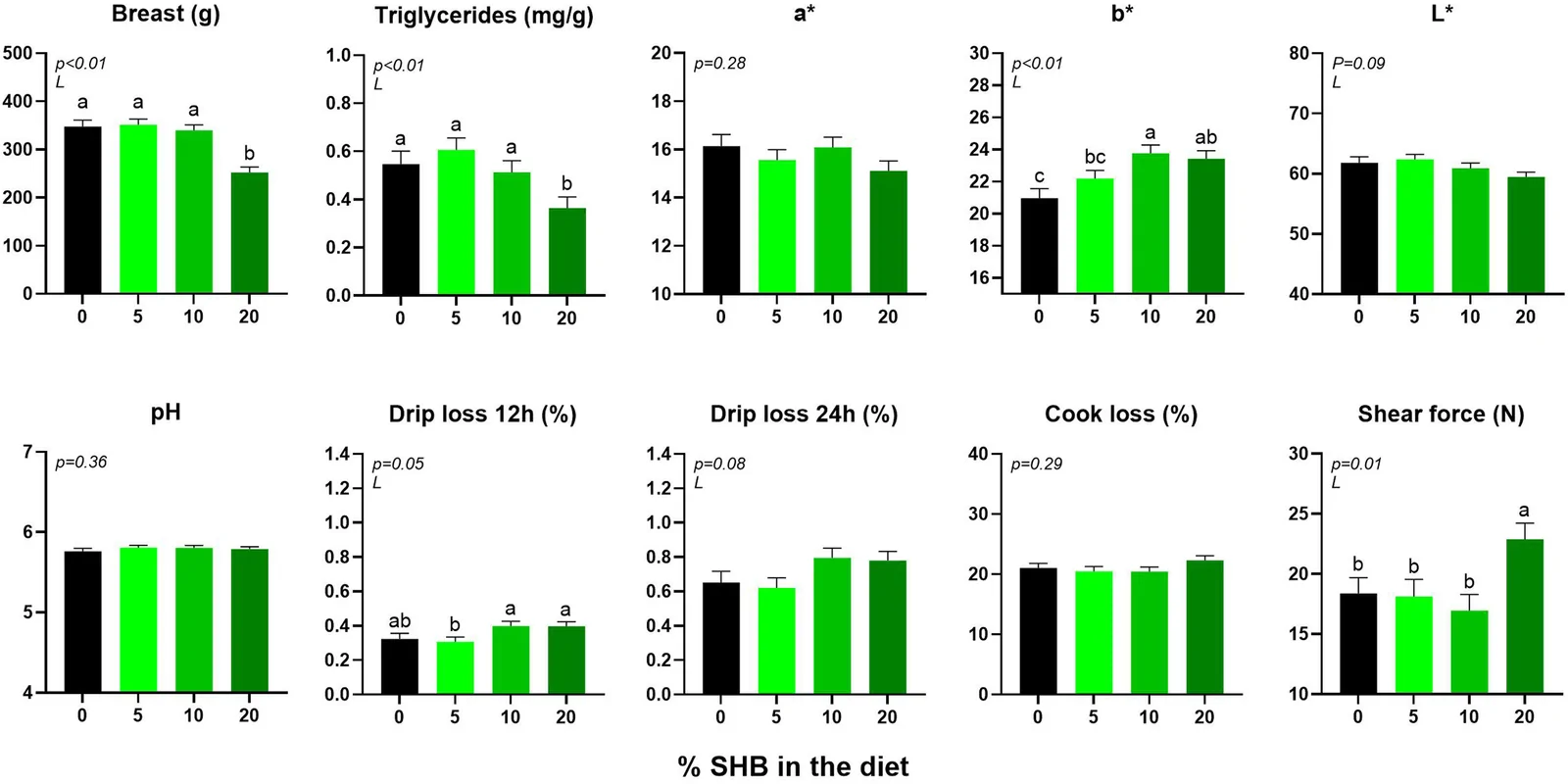
Breast weight, triglyceride content, and quality of the meat in broiler chickens receiving 0, 5, 10, or 20% dietary spent hemp biomass (SHB). Letters denote statistical differences (p<0.05) between groups. The p-value reported in the graph is the overall treatment effect. *L* denotes a significant (p<0.05) linear effect of the SHB dose.

Meat quality attributes of *pectoralis major* for birds fed with or without SHB are shown in Figure 4. The pH, cooking loss, and a* (redness) were not affected by feeding SHB. With regard to instrumental color, b* values (yellowness) increased linearly with the amount of SHB in the diet, with higher values in the SHB10 and SHB20 groups compared to CON. L* values (lightness) decreased linearly with the level of SHB in the diet and had a tendency (*P* = 0.09) to be affected by feeding SHB. Drip loss of the breast increased linearly with the level of SHB in the diet, especially for SHB10 and SHB20. The SHB20 breast samples had higher shear force (i.e., lower tenderness) values compared to all other groups. The yellowness of the meat (i.e., b*) and the pH were higher in males compared to females, but none of the parameters were differently affected between sexes by feeding SHB (Figure S5 in Supplementary File).

### Fatty acid profile of the breast tissue

Results of the FA proportions (as % of annotated FAs) in *pectoralis major* are available in Table 2. Dietary inclusion of SHB had a significant impact on the proportion of several FAs in breast tissue, especially in SHB20 birds. Feeding 20% SHB increased the proportion of saturated FAs (especially C18:0) and bacteria-derived FAs (especially C17:0) compared to the lower doses of SHB, while decreasing the proportion of unsaturated FAs, including mono- and polyunsaturated FAs (**PUFA**), most notably C18:3n3 and C18:2n6, compared to the animals fed 5 or 10% SHB, but was not different than the control group. Moreover, dietary SHB inclusion up to 20% also increased the proportion of FAs detrimental to cardiovascular health, as indicated by the atherogenic index, the index of atherogenicity, and the hypocholesterolemic/hypercholesterogenic ratio.

**Table 2:** Fatty acid profile as a percentage of total annotated fatty acids in the *pectoralis major* of broilers fed different amounts of dietary spent hemp biomass.

	**% spent hemp biomass**							
**Fatty acid**	**0**	**5**	**10**	**20**	***SEM***	**P-value**	**L**	**Q**
C12:0	0.035	0.042	0.031	0.040	*0.006*	*0.40*	*0.85*	*0.67*
C13:0	1.24	0.75	0.91	0.99	*0.189*	*0.22*	*0.98*	*0.12*
C14:0	0.56	0.55	0.51	0.74	*0.116*	*0.26*	*0.10*	*0.27*
C14:1	0.053	0.057	0.052	0.062	*0.010*	*0.76*	*0.44*	*0.69*
C15:0	0.55	1.04	1.04	1.16	*0.411*	*0.68*	*0.35*	*0.53*
C16:0	16.8	17.0	17.1	18.3	*0.613*	*0.10*	*0.02*	*0.46*
C16:1	0.96^ab^	1.44^a^	1.49^a^	1.15^b^	*0.141*	*<0.01*	*0.68*	*<0.01*
C17:0	0.27^b^	0.25^b^	0.30^b^	0.56^a^	*0.086*	*<0.01*	*<0.01*	*0.16*
C17:1	0.066	0.111	0.109	0.103	*0.031*	*0.68*	*0.62*	*0.32*
C18:0	8.58^ab^	8.11^b^	8.22^b^	9.72^a^	*0.508*	*0.01*	*<0.01*	*0.05*
C18:1trans^1^	0.17	0.33	0.28	0.28	*0.080*	*0.50*	*0.72*	*0.27*
C18:1n9c	17.2^ab^	19.0^a^	18.5^a^	15.9^b^	*0.778*	*<0.01*	*<0.01*	*<0.01*
C18:1n15t,10c	2.02^ab^	1.85^b^	1.93^b^	2.24^a^	*0.112*	*0.01*	*<0.01*	*0.04*
C18:2n6c	30.7^ab^	33.4^a^	32.7^a^	28.7^b^	*1.511*	*0.02*	*0.02*	*0.03*
C20:0	0.14	0.19	0.16	0.22	*0.035*	*0.20*	*0.10*	*0.81*
C18:3n6	0.31	0.32	0.24	0.36	*0.070*	*0.48*	*0.57*	*0.29*
C18:3n3	3.45^a^	3.89^a^	3.75^a^	2.33^b^	*0.326*	*<0.01*	*<0.01*	*<0.01*
C20:2	0.80^ab^	0.60^b^	0.75^ab^	0.87^a^	*0.077*	*<0.01*	*0.02*	*0.08*
C22:0	0.74	0.71	0.85	0.93	*0.119*	*0.21*	*0.05*	*0.92*
C20:3n6	0.31^b^	0.54^ab^	0.38^b^	0.76^a^	*0.139*	*0.02*	*0.01*	*0.48*
C22:1n9	0.36	0.30	0.28	0.43	*0.115*	*0.58*	*0.33*	*0.31*
C23:0	6.41^ab^	5.17^b^	5.68^ab^	6.75^a^	*0.558*	*0.04*	*0.07*	*0.04*
C20:4n6	0.52	0.30	0.31	0.48	*0.141*	*0.42*	*0.61*	*0.12*
C24:0	0.86	0.78	0.60	1.17	*0.245*	*0.09*	*0.08*	*0.08*
C20:5n3	0.91	0.67	0.79	0.85	*0.294*	*0.88*	*0.81*	*0.63*
C24:1n9	1.82	1.68	1.66	1.98	*0.257*	*0.54*	*0.28*	*0.30*
C22:6n3	1.24^b^	0.87^c^	0.98^bc^	1.55^a^	*0.117*	*<0.01*	*<0.01*	*<0.01*
SAT	37.4^b^	34.6^b^	35.8^b^	41.3^a^	*1.811*	*<0.01*	*<0.01*	*0.02*
UFA	62.6^ab^	65.4^a^	65.4^a^	58.7^b^	*1.664*	*<0.01*	*<0.01*	*<0.01*
MUFA	23.7^ab^	24.6^a^	24.2^a^	22.4^b^	*0.708*	*0.02*	*0.01*	*0.06*
PUFA	38.9^ab^	40.8^a^	41.0^a^	36.2^b^	*1.434*	*<0.01*	*<0.01*	*0.01*
Bacteria	8.54^ab^	7.31^b^	7.68^b^	9.66^a^	*0.823*	*0.04*	*0.03*	*0.05*
ω3	6.01	5.43	5.53	4.91	*0.404*	*0.16*	*0.04*	*0.92*
ω6	32.1^ab^	34.7^a^	33.7^a^	30.4^b^	*1.372*	*0.02*	*0.03*	*0.03*
ω6/ω3	5.78	6.48	6.18	6.40	*0.392*	*0.48*	*0.49*	*0.53*
PUFA/SAT	1.07^ab^	1.19^a^	1.16^a^	0.89^b^	*0.091*	*0.01*	*<0.01*	*0.04*
AI^2^	0.31^b^	0.30^b^	0.30^b^	0.37^a^	*0.020*	*<0.01*	*<0.01*	*0.07*
h/H^3^	3.33^ab^	3.55^a^	3.44^a^	2.86^b^	*0.202*	*<0.01*	*<0.01*	*0.06*
IA^4^	0.28^b^	0.27^b^	0.28^b^	0.33^a^	*0.017*	*<0.01*	*<0.01*	*0.11*

Dissimilar letters denote statistical differences between groups when the overall treatment effect was significant at P ≤ 0.05.

1 Sum of all trans 18:1

2 Atherogenic Index = [C12:0 + 4 × C14:0 + C16:0)/(MUFA + PUFA)] (Fusaro et al., 2019)

3 Hypocholesterolemic/hypercholesterolemic ratio = [(C18:1n9c + C18:1trans + C18:1n15t,10c + C18:2n6 + C18:3n6 + C18:3n3 + C20:3n6 + C20:4n6 + C20:5n3 + C22:6n3)/(C14:0 + C16:0)] (Fernández et al., 2007)

4 Index of atherogenicity= [(C12:0+ C14:0 × 4 + C16:0)/(C18:1n9c + C18:1trans + C18:1n15t,10c + C18:2n6 + C18:3n6 + C18:3n3 + C20:3n6 + C22:1n9 + C20:4n6 + C20:5n3 + C24:1n9 + C22:6n3)] (Ulbricht and Southgate, 1991)

Data on total FA in breast tissue (μg/g) (Table S4 in Supplementary File) indicated a significant linear decrease in the amount of FAs in the breast of broilers fed SHB. Overall, SHB20 breast samples possessed the lowest amounts of most FAs, except C14:0, bacteria-derived FAs (C15:0, C17:0, C17:1), C22:0, and a few of the minor unsaturated FAs such as C18:1n10t, C22:1n9, and C20:5n3.

### Internal organs

SHB affected the absolute weight of a few internal organs, with linear decreases in the weights of the gizzard and the spleen as the dose of SHB increased (Figure 5). When corrected by BW, increases in dietary SHB linearly increased the relative weight (g/kg BW) of the brain and the liver; however, the relative size of the spleen was negatively affected by the increased dose of SHB in the diet (Figure 5). When considering the sex of the animals (Figure S6 in Supplementary File), feeding SHB did not affect the size of the organ differently between males and females. However, the heart and the liver were larger, while the brain tended to be larger (*P*=0.08) in males vs. females, but females had a proportionally larger spleen compared to males.

**Figure 5:**
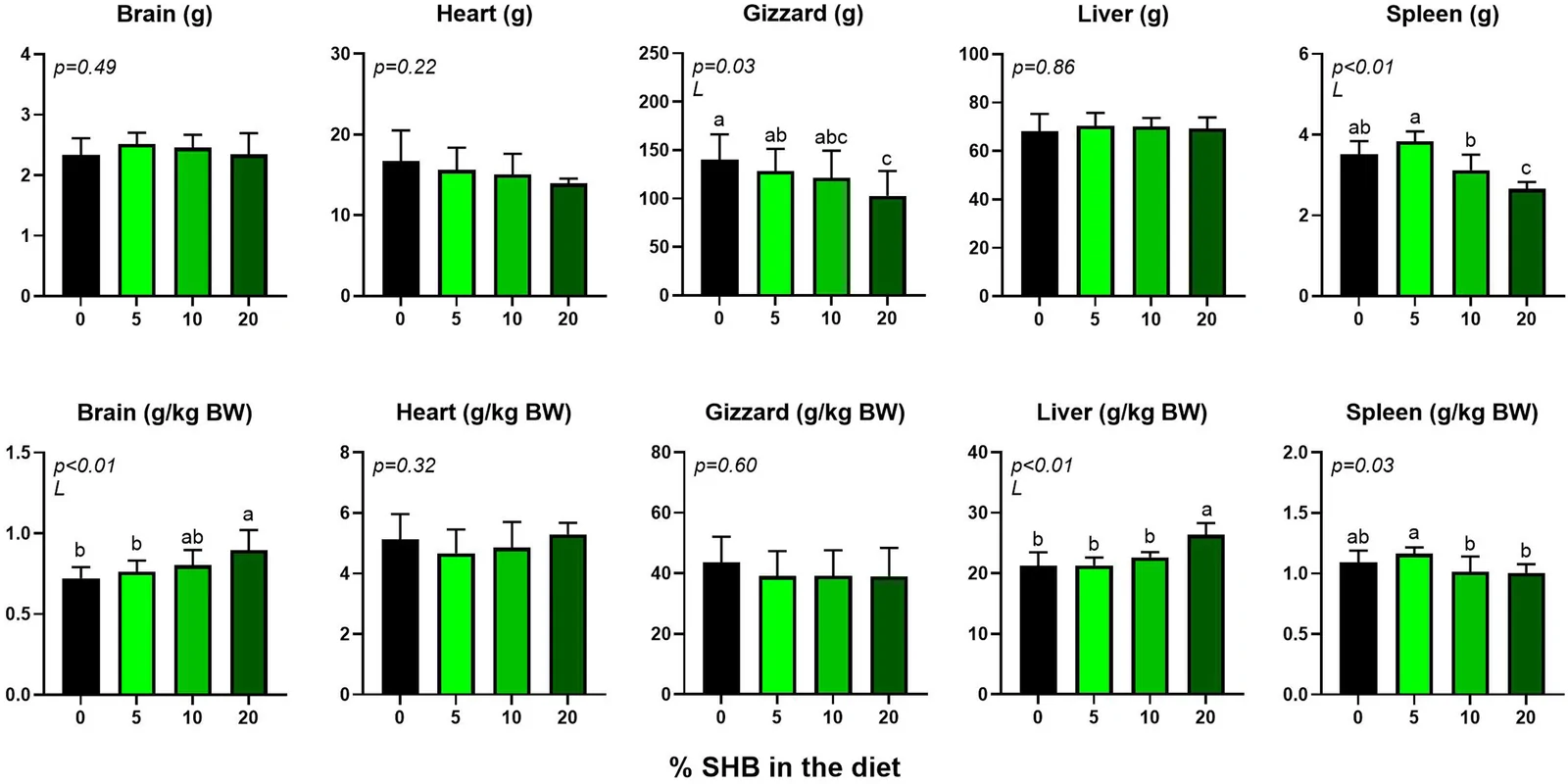
Internal organ weights, expressed in weight (g) and percentage of body weight (g/kg BW) of broilers fed diets containing 0, 5, 10, or 20% spent hemp biomass (SHB), respectively. *L* denotes a significant (p<0.05) linear effect of the SHB dose.

### Gene expression in the adipose tissue

The effect of feeding SHB on the transcription of genes coding for proteins involved in lipogenesis and fatty acid oxidation is reported in Figure 6. The analysis indicated an increase in fatty acid transport (i.e., *FABP4*) and oxidation of FA in the peroxisomes (i.e., *ACAA1* and *ACOX1*) with the dose of SHB received, but this was only significant with 20% dietary SHB. This effect was only observed in male birds and not in female birds, where no significant effects were found (Figure S7 in Supplementary File). *ACSL1*, coding for a protein involved in the activation of FAs, was downregulated by feeding 5% SHB. The transcription of *FABP4, LPL*, and *ACOX1* was overall higher in males than in females (Figure S7 in Supplementary File).

**Figure 6:**
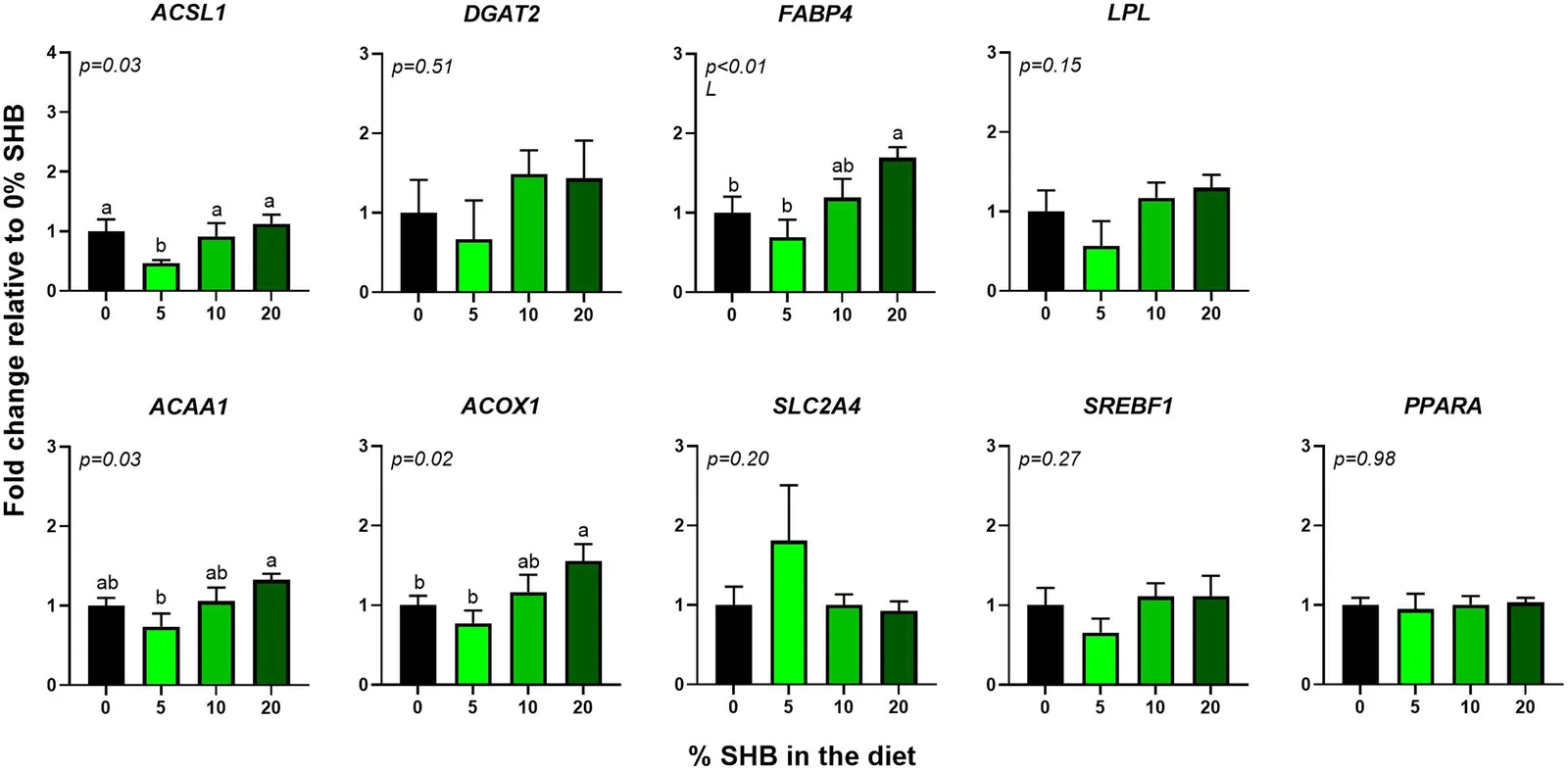
Transcription of genes related to lipogenesis and lipid oxidation in the internal adipose tissue of broilers fed diets containing 0, 5, 10, or 20% spent hemp biomass (SHB), respectively. *L* denotes a significant (p<0.05) linear effect of the SHB dose.

### Corticosterone

The corticosterone in the feathers increased linearly with the dose of SHB in the diet, with significantly larger values in the birds receiving 20% SHB compared to the other groups, and no difference between the sexes (Figure 7).

**Figure 7:**
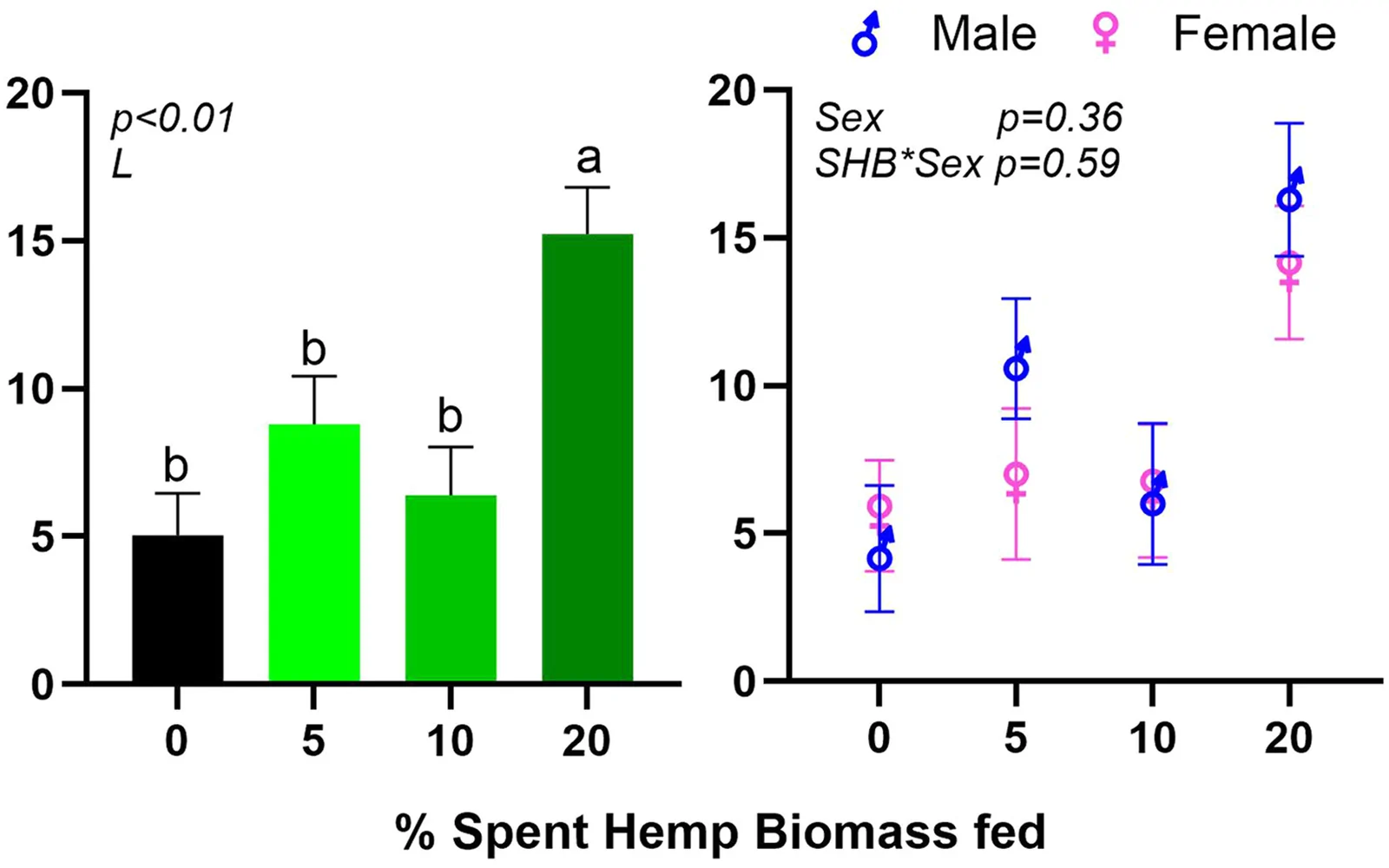
Corticosterone in the feathers of broilers fed diets containing 0, 5, 10, or 20% spent hemp biomass (SHB), respectively. *L* denotes a significant (p<0.05) linear effect of the SHB dose.

## Discussion

### Elevated dietary SHB detrimentally impacts performance

In our experiment, the inclusion of dietary SHB ≥10% in broiler rations had negative consequences on bird performance as indicated by FCR, BW, and breast weight data, while intake was not affected. Previous studies utilizing dietary hempseed (Mahmoudi et al., 2015) and hempseed oil (Kanbur, 2022) in broiler rations reported decreases in weight gain and overall performance, respectively. However, other studies did not find any differences in performance parameters in birds fed hempseed (Vispute et al., 2019) and hempseed oil (Jing et al., 2017), while other researchers have reported that dietary hempseed improved BW over control birds (Khan et al., 2010; Parr et al., 2020; Skřivan et al., 2020). The aforementioned studies primarily utilized hempseed and hempseed derivatives, which are markedly higher in fat and protein and lower in fiber compared to SHB used in the present study (Burton et al., 2022) and likely account for the differences observed. Sopian et al. (2024) noted that birds fed *cannabis* residue at 0.5, 1, and 2% in the diet experienced lower feed intake than control birds, but no differences in performance. These findings are of interest, provided *cannabis* residues fed were similar in composition to SHB.

Performance differences despite similar feed intake may be attributed to higher numerical NDF and ADF observed in SHB diets compared to CON. Increased levels of fiber in poultry diets have been linked to inhibited growth and performance, namely due to decreases in nutrient absorption and digestibility (Singh and Kim, 2021). As denoted by FCR, SHB20 birds experienced significant deterioration in feed conversion, especially during the first 20 days of the trial. These findings are in alignment with Šťastník et al. (2015), who observed significant decreases in BW and an increase in FCR as dietary hempseed cake increased in broiler rations, likely due to the content of ADF in hempseed cake. In our experiment, fiber (measured as NDF) was consistently 12% higher in all three diets of SHB groups. If the level of fiber affected performance, this should be consistent among all birds receiving SHB, but only birds receiving>5% SHB had a decrease in performance; that was worst for birds receiving 20% SHB. Thus, the observed difference in the level of fiber, fat, or CP in the diets cannot explain the effect on performance. Moreover, metabolizable energy and amino acid values were similar across all treatments, further supporting the idea that diet composition cannot account for performance differences. The present study did not measure digestible energy, making it difficult to ascertain the SHB effect on nutrient absorption and utilization. Findings by Sopian et al. (2024) utilizing *cannabis* residue in broiler diets demonstrated improvements in the ileal villus height/crypt depth ratio at inclusion up to 1% in the diet, indicating positive effects on intestinal morphology and digestive parameters. Despite these findings, comparisons to the present study may be hard to draw, given the relatively low inclusion levels of *cannabis* residues (0.5, 1, and 2%, respectively) fed in the study of Sopian et al. (2024).

Diets in our experiment differed in the levels of minerals, with a linear increase in most minerals as the level of SHB increased in the diet. Among all mineral-related parameters, the ratio of Ca/P is known to significantly affect the performance of the broilers, with a generally recognized optimal Ca/P ratio of 2. When the ratio is excessive, the higher Ca level can compromise the absorption of P, negatively affecting performance (David et al., 2023). Jabbar et al. (2024) observed a significant decrease in performance with a Ca/P ratio of 2.3, with a decrease in feed intake. In our experiment, the diet of the SHB20 group had a Ca/P ratio of 2.4; however, the feed intake was not affected.

Antinutritional compounds likely did not impact performance parameters, as there were no differences in feed intake and the absence of detectable toxins in the SHB used in the study (Parker et al., 2022). The effect of cannabinoids and other organic compounds included in SHB should not be ruled out, provided these compounds activate gene receptors in the body, which can modify gastrointestinal function and enzymatic activity in broilers (Bialkowski et al., 2023). SHB used in the present study possessed elevated concentrations of cannabinoids, namely CBD and cannabidiolic acid, which accounted for a combined 74.3% of total cannabinoids (Parker et al., 2022). Cannabinoid activation of endocannabinoid receptors in the gastrointestinal system may decrease intestinal motility and secretion, while also impacting peristalsis and intestinal barrier permeability (Martínez et al., 2020; Crowley et al., 2024). Therefore, it is plausible that cannabinoids in SHB have an unknown effect on broiler metabolic activity and nutrient utilization. Moreover, a synergistic effect of elevated dietary cannabinoids and fiber in growing broilers may also exist.

The changes observed in gene expression were primarily sex effects, except for *FABP4*, which indicates an increase in fatty acid transport and, by the increase in expression of *ACAA1* and *ACOX1*, the oxidation of FA in the peroxisomes. All three genes are targets of the Peroxisome Proliferator-activated Receptor (**PPAR**) (Rakhshandehroo et al., 2010). Cannabinoids are known to activate PPAR, particularly CBD and CBG (O'Sullivan, 2016; D'Aniello et al., 2019). Furthermore, several of the FABPs, including FABP4, have been indicated to play active roles in the hepatic transport of cannabinoids (Elmes et al., 2015). The above results are indicative of a possible higher catabolic state in the adipose tissue of the birds fed the highest level of SHB; however, they cannot explain the obviously smaller size of those birds. It is possible that SHB20 birds also had a lower capacity to utilize the feed. Thus, further research is warranted to examine SHB effects on intestinal morphology and function to elucidate the growth differences observed in the present study. Prior data indicated that a low dose of spent hemp biomass (2%) increases the size of intestinal villi (Sopian et al., 2024). Thus, it is possible that a higher dose of SHB, as in our study, was detrimental to feed absorption, as partially suggested by the increase in frequency and severity of pasty vent. The lower absorption of feed is also partly supported by the corticosterone data, as prior data indicated that birds on a restrictive feed availability had a higher level of plasma corticosteroid than those being fed *ad libitum* (Yan et al., 2021).

Increased incidence of pasty vent has been associated with decreased digestibility of the diet (Roy et al., 2010), but, more likely, is associated with a change in the microbiota of the ceca (Swayne et al., 1992; Cesare et al., 2017). Enrichment of the bacteria-derived FAs in the breast could support dysbiosis of the microbiota of the gut fed 20% SHB in our experiment.

### SHB alters organ growth

Inclusion of SHB in broiler diets demonstrated a clear impact on the weights of several organs. The most profound effect was observed in birds fed SHB at 20%, with significant differences in absolute weight (spleen and gizzard) and relative proportions (increased brain and liver and decreased spleen). Previous studies have noted that birds supplemented with dietary hemp seed experienced significant alterations in their organ weights in broilers (Khan et al., 2009; Mahmoudi et al., 2012) and quail (Konca et al., 2014). Feeding an increased dose of hempseed cake to broilers resulted in decreased liver weight (Mahmoudi et al., 2012; Konca et al., 2014). Sopian et al. (2024) also noted a trend towards lower liver weight proportions in broilers via stepwise increases in *cannabis* residues. However, Khan et al. (2009) found dietary hemp seed at 20% inclusion resulted in higher total liver weight compared to 0 and 5% hemp seed treatment.

Previous studies utilizing SHB (Parker et al., 2022) and hemp stubble (Krebs et al., 2021) in lamb diets did not find significant differences in organ weights of sheep fed hemp versus control diets. However, liver weights in hemp-fed animals were numerically higher compared to controls in both studies. The stronger effect on birds vs. ruminants may be attributed to species differences in mammalian and avian physiology (Ricklefs et al., 1996; Scanes, 2020), in addition to ruminant and monogastric metabolic variations (Apajalahti, 2005). Nevertheless, the role of SHB and associated compounds cannot be dismissed to explain the differences between studies. Lambs in our prior study fed 20% SHB consistently consumed 150 mg CBD/kg BW, while the birds in the present study, consuming the same dose of SHB, ingested 176 mg CBD/kg BW on average, but during the first 3 weeks in the experiment, ingested >200 mg/kg BW. Animal models have demonstrated that dietary supplementation of cannabinoids led to increases in liver weight in primates (Rosenkrantz et al., 1981), humans (Larson et al., 2025), rats, and dogs (Gingrich et al., 2023) due to hepatotoxicity. This is partly due to the effect of the cannabinoids on the transcriptome of the liver, although the effect is minor (Irawan and Bionaz, 2024).

Data regarding brain weight in the present study are also of interest, suggesting dietary SHB has a dose-dependent effect on the proportion of brain size to body weight. CBD intake has been linked to increases in regional hippocampal blood flow (Bloomfield et al., 2020) and volume (Beale et al., 2018). Thus, it is possible that CBD obtained from SHB used in the present study had a morphological effect on brain volume in the growing birds.

### High SHB in diet impacts meat quality

Dietary SHB, especially at inclusion levels of 20% in the diet, negatively affected meat quality. Concerning instrumental color, higher b* (yellowness) values observed in SHB10 and SHB20 groups could indicate lower heme pigmentation and concentration of oxymyoglobin in these groups. Previous studies found no difference in color space values of meat in quail supplemented with hemp oil (Göçmen and Kanbur, 2021) and broilers fed hempseed (Tufarelli et al., 2023). However, Kanbur (2022) reported significant reductions in breast meat yellowness in broilers supplemented with high levels of hempseed oil versus control birds. It has been reported that an increase in b* in meat is largely due to differences in myoglobin redox forms (Bennato et al., 2020) namely, oxidation of oxymyoglobin to metmyoglobin (Macdougall, 2010). This may indicate increased oxidation in SHB10 and SHB20 breast samples. However, oxidative parameters (e.g., TBARS) were not measured in the present study to confirm differences in oxidation.

It is possible that ≥10% SHB inclusion led to post-mortem losses in protein solubility, as indicated by significant increases in drip loss in SHB10 and SHB20 birds compared with SHB5. Post-mortem fluid loss in muscle is correlated with the concentration of undenatured, soluble protein, with higher concentrations of sarcoplasmic protein in poultry meat linked to increases in water-holding capacity (Bowker and Zhuang, 2013; Bowker and Zhuang, 2015). High drip loss in SHB10 and SHB20 may also be a consequence of lower muscle triglyceride content compared to SHB5, as the content of triglycerides is an indicator of intramuscular fat in chicken meat (Cui et al., 2022). It has been reported that higher intramuscular fat content is correlated with lower drip loss (Czarniecka-Skubina et al., 2010) and may explain differences observed in the present study.

Dietary SHB up to 20% resulted in tougher meat. Interestingly, lambs fed SHB up to 20% in the diet also experienced significant decreases in meat tenderness (Parker et al., 2022). Lower TG levels in SHB20 breasts may have also led to decreases in tenderness, since fat deposition within muscle has been shown to increase tenderness in chicken meat (Cui et al., 2022).

### SHB inclusion alters lipid profile in breast tissue

Dietary SHB resulted in modifications to FAs in breast tissue, most notably at 20% inclusion. These results are surprising, given the lack of differences in the proportion of FAs in the rations. Of particular interest is the increase in the proportion of odd-chain FAs in the breast, mostly derived from bacteria (Qin et al., 2023). As also indicated above, this finding suggests an effect of SHB on gut microbiota, especially at high SHB dosage in the diet. Cannabinoids, especially CBD, have been shown to alter gut microbiota while modifying the FA profile in chickens (Konieczka et al., 2022). CBD found in SHB may partially explain our results.

Breast samples from SHB20 had a lower amount of TG and FAs, suggesting overall lower deposition of fat in carcass tissues, indicating that SHB feeding at 20% impairs fat synthesis and deposition. Because dietary composition and feed intake did not differ among treatments, the TG and FA results suggest a metabolic effect of SHB. It has been demonstrated that activation of the endocannabinoid receptor system by CBD results in a downregulation of lipogenesis and FA accumulation in intramuscular fat depots of skeletal muscle (Bielawiec et al., 2023). Although we did not measure gene transcription in the pectoralis major, our data on transcription of fat metabolism-related genes in abdominal fat support lower triglyceride accumulation, but not via reduced lipogenesis; rather, via increased FA catabolism in peroxisomes. However, those data should be taken cautiously, as adipose tissue and muscle are two different tissues.

SHB at 20% in the diet resulted in lower PUFA in breast, notably total ω-6 and 18:3ω-3, while SFA were significantly higher. These findings conflict with previous monogastric trials utilizing dietary hempseed oil, in which greater enrichment of PUFA was observed in pig meat (Mourot and Guillevic, 2015), egg yolks (Gakhar et al., 2012), and broiler tissues (Jing et al., 2017). Tufarelli et al. (2023) also reported greater PUFA in broiler meat tissues from birds fed hempseed cake. However, the FA profile of hemp seed and hemp oil differs from that of SHB; furthermore, the FAs were balanced between the various diets in our experiment. Thus, the effect observed appears to be determined more by the level of SHB in the diet than the FA composition of the diets.

Overall, feeding 20% SHB had a detrimental influence on the health profile of broiler meat, as denoted by an increase in saturated FAs and a decrease in PUFA, with a concomitant increase in FAs associated with atherogenicity (Chen and Liu, 2020); however, feeding 5 and 10% SHB did not affect the FA profile or the quality of broiler meat.

### Limitations

Our study has several limitations. The corticosterone and TG assays were based on prior validated methods and commercial kits; however, we did not fully validated the assays, except for reporting the intra-assay variation. We did not measure CBD in the rations; thus, CBD intake was only estimated. Although cannabinoid levels in edible tissues are important for the legalization of SHB as feed for broilers, we were unable to obtain those data. The lack of histological analysis of the liver and brain limited the reliability of the interpretations of the observed effects of feeding 20% SHB on the weight of those organs relative to the BW. The interpretation of the pasty vent as gut dysbiosis should be taken with caution, as no microbiota analysis was performed to validate it.

## Conclusions

Adding SHB to broiler diets up to 5% may be used without adverse effects on bird performance, meat quality, or nutritional profile. However, dietary supplementation ≥10% may lead to performance losses, especially in FCR during the early stages of the broiler’s life cycle, affecting finished weight and detrimentally impacting meat quality parameters, particularly water-holding capacity and tenderness. Fat metabolism and FA deposition are also altered by >5% SHB inclusion in the diet. Our data indicated a possible negative effect of SHB in the diet on the gut microbiota of broilers and, at doses >10%, higher corticosterone in feathers as a possible indicator of chronic stress, affecting fat metabolism in adipose tissue at the transcriptomic level, especially in male birds. Overall, our data indicate that feeding up to 5% SHB in the diet is safe for broiler chickens, but higher doses are not recommended.

## Author Contributions

**Massimo Bionaz**: conceived and designed the work with Serkan Ates and James Hermes; curated the information and supporting materials; acquired funding with Serkan Ates; administered and supervised the project with James Hermes; contributed to the investigation and validation; prepared the visual materials; provided resources; and drafted the manuscript with Nathan Parker. **Serkan Ates**: contributed to the conception and design of the work, funding acquisition, investigation, interpretation of the evidence, and critical intellectual revision of the manuscript. **James Hermes**: contributed to the conception and design of the work, development of the methodological approach, project supervision, investigation, interpretation of the evidence, and critical intellectual revision of the manuscript. **Nathan Parker**: contributed to the methodological approach of the analysis of the meat, acquisition and investigation of the relevant evidence, formal analysis and interpretation, and validation of the work; and drafted the manuscript with Massimo Bionaz. **Agung Irawan**: contributed to the methodological approach of the fatty acid analysis, acquisition and investigation of the relevant evidence, formal analysis and interpretation, and critical intellectual revision of the manuscript. **Carolyn Pearce**: contributed to the methodological approach of the RTqPCR, acquisition and investigation of the relevant evidence, formal analysis and interpretation, and critical intellectual revision of the manuscript. **Dresen Ferschweiler**: contributed to the methodological approach of the corticosterone analysis, acquisition and investigation of the relevant evidence, formal analysis and interpretation, and critical intellectual revision of the manuscript.

## Ethics declaration

This study was conducted in accordance with the ARRIVE (Animal Research: Reporting of In Vivo Experiments) guidelines. This study was approved by the Oregon State University (OSU) Animal Care and Use Committee. (Approval No. ACUP# 0192)

## Disclosures

The authors declare that there are no conflicts of interest.
