# Supplementary material for: Spent hemp biomass in broiler diets: effects on performance, meat quality, expression of genes in internal adipose tissue, and corticosterone in feathers
Source: Poult Sci. 2026 Aug 28;105(12):107692. doi: 10.1016/j.psj.2026.107692 (PMC13634519; doi:10.1016/j.psj.2026.107692)
Supplement: Supplementary file 1 [file mmc1.docx]

**Table S1.** Primer pairs used for the RTqPCR analysis**.**

| Symbol | Name | Accession | Forward | | Reverse | | Amplicon |
| --- | --- | --- | --- | --- | --- | --- | --- |
|  |  |  | ***St.*** | ***Sequence*** | ***St.*** | ***Sequence*** |  |
| ACAA1 | Acetyl-CoA acyltransferase 1 | NM_001197288.1 | 364 | TATCCCAGAGACGGTGCCTT | 454 | ATTTCGGATGCCACCAGCTA | 91 |
| ACOX1 | Acyl-CoA oxidase 1, palmitoyl | NM_001006205.1 | 599 | GCCAGGTGGACTTGGAAAGA | 705 | GCTGCCGTATAGGAACAATGAAG | 107 |
| ACSL1 | Acyl-CoA synthetase long-chain family member 1 | NM_001012578.1 | 1839 | GAGAGCTTGCAGGCCTTCTTAC | 1941 | ATAGCTTCTCATATGTGCCTTCAAATC | 103 |
| DGAT2 | diacylglycerol o-acyltransferase 2 | XM_419374 | 928 | ATTGGCTTTGCTCCATGCAT | 1027 | CGCCCACAACAGTGGTGAT | 100 |
| FABP4 | Fatty acid binding protein 4 | NM_204290.2 | 137 | GGGGTTTGCTACCAGGAAGATG | 412 | CATTCCACCAGCAGGTTCCC | 276 |
| GAPDH | Glyceraldehyde-3-phosphate dehydrogenase | NM_204305.1 | 194 | AATATGATTCTACACACGGACACTTCA | 293 | TCACGCTCCTGGAAGATAGTGA | 100 |
| LPL | Lipoprotein Lipase | NM_205282.2 | 1573 | AGGAGAAGAGGCAGCAATA | 1794 | AAAGCCAGCAGCAGATAAG | 222 |
| MRPL39 | Mitochondrial ribosomal protein L39 | BX936152 | 69 | AATGGCGCAGGTTCAACTCT | 168 | TGCCTCTCCTTCTCTTTGGTAAA | 100 |
| MRPS9 | Mitochondrial ribosomal protein s9 | NM_001277758.1 | 907 | AGGCTGGAGCGATACGTTTG | 996 | GCAGCCCAGCTTGTCTCATAA | 90 |
| PPARA | Peroxisome Proliferator-activated Receptor Alpha | NM_001001464 | 1553 | GTGGAGATCGTCCTGGTCTTG | 1652 | TCAGGATGGTTGGTTTGCAA | 100 |
| SLC2A4 | Solute carrier family 2, facilitated glucose transporter member 4 | XM_025142445.1 | 330 | CATGATTGCCACCAACGCC | 432 | GAGCAGGAACCGACCAATGA | 103 |
| SREBF1 | Sterol regulatory element binding transcription factor 1 | NM_204126.2 | 1058 | GCGCTACCGCTCATCCAT | 1150 | CTCAGGATCGCCGACTTGTT | 93 |
| UXT | Ubiquitously expressed transcript | BX950253 | 14 | GGCGGCGATAACATGCAT | 118 | GCTCTCTGCGCTTTGATTCAA | 105 |

**Table S2.** Amino acid profile (w/w %) of experimental broiler dietary rations fed 0 (control), 5, 10, or 20% spent hemp biomass.

|  | **Spent hemp biomass** | | | |
| --- | --- | --- | --- | --- |
| **Component** | **0%** | **5%** | **10%** | **20%** |
| Alanine | 0.96 | 0.96 | 0.99 | 0.93 |
| Arginine | 1.35 | 1.32 | 1.37 | 1.28 |
| Aspartic acid | 2.17 | 2.17 | 2.34 | 2.26 |
| Cysteine | 0.28 | 0.27 | 0.29 | 0.27 |
| Glutamic acid | 3.81 | 3.67 | 3.84 | 3.45 |
| Glycine | 0.85 | 0.85 | 0.89 | 0.86 |
| Histidine | 0.49 | 0.48 | 0.5 | 0.46 |
| Isoleucine | 0.83 | 0.76 | 0.84 | 0.81 |
| Leucine | 1.63 | 1.57 | 1.64 | 1.54 |
| Lysine | 1.15 | 1.11 | 1.21 | 1.09 |
| Methionine | 0.36 | 0.36 | 0.39 | 0.38 |
| Phenylalanine | 0.97 | 0.95 | 1.01 | 0.95 |
| Proline | 1.14 | 1.11 | 1.18 | 1.12 |
| Serine | 1.05 | 1.03 | 1.06 | 0.97 |
| Threonine | 0.75 | 0.74 | 0.79 | 0.72 |
| Tryptophan | 0.27 | 0.27 | 0.28 | 0.28 |
| Tyrosine | 0.67 | 0.67 | 0.7 | 0.65 |
| Valine | 0.93 | 0.89 | 0.94 | 0.96 |
| AA (%N) | 82.3 | 74.6 | 81.6 | 76.8 |

**Table S3.** Fatty acid composition (relative %) of the experimental broiler dietary rations containing either 0 (control), 5, 10, or 20% spent hemp biomass.

|  | **Spent Hemp biomass** | | | | | | |  |
| --- | --- | --- | --- | --- | --- | --- | --- | --- |
| **Fatty acid** | **0%** | **5%** | | **10%** | | **20%** | |  |
| C14:0 | 0.10 | | 0.19 | | 0.12 | | 0.16 | |
| C15:0 | 0.01 | | 0.03 | | 0.02 | | 0.06 | |
| C16:0 | 11.8 | | 11.8 | | 11.7 | | 12.1 | |
| C16:1 | 0.12 | | 0.12 | | 0.13 | | 0.11 | |
| C17:0 | 0.01 | | 0.08 | | 0.08 | | 0.02 | |
| C18:0 | 2.76 | | 3.41 | | 3.37 | | 3.31 | |
| C18:1c9 | 23.0 | | 22.2 | | 21.7 | | 20.6 | |
| C18:2c9t11 | 0.35 | | 0.31 | | 0.22 | | 0.21 | |
| C18:2n6c | 53.4 | | 53.1 | | 53.8 | | 53.6 | |
| C18:2t10c12 | 0.04 | | 0.13 | | 0.03 | | 0.05 | |
| C18:3n3 alpha | 7.19 | | 7.48 | | 7.56 | | 8.30 | |
| C18:3n6 gamma | 0.46 | | 0.46 | | 0.51 | | 0.52 | |
| C20:0 | 0.09 | | 0.10 | | 0.07 | | 0.09 | |
| C20:3n3 | 0.01 | | 0.03 | | 0.02 | | 0.02 | |
| C20:4n6 | 0.01 | | 0.02 | | 0.03 | | 0.02 | |
| C20:5n3 | 0.03 | | 0.02 | | 0.04 | | 0.04 | |
| C21:0 | 0.18 | | 0.15 | | 0.20 | | 0.20 | |
| C22:5 | 0.01 | | 0.02 | | 0.08 | | 0.08 | |
| C22:6n3 | 0.10 | | 0.04 | | 0.04 | | 0.04 | |
| C23:0 | 0.06 | | 0.08 | | 0.07 | | 0.08 | |
| C24:0 | 0.18 | | 0.18 | | 0.22 | | 0.26 | |
| C24:1n9 | 0.14 | | 0.07 | | 0.04 | | 0.06 | |

**Table S4.** Fatty acid profile as μg/g of *pectoralis* major of broilers fed with different amounts of dietary spent hemp biomass.

|  | **% spent hemp biomass** | | | |  |  |  |  |
| --- | --- | --- | --- | --- | --- | --- | --- | --- |
| **Fatty acid** | **0** | **5** | **10** | **20** | ***SEM*** | **P-value** | **L** | **Q** |
| C12:0 | 15.9^ab^ | 18.1^a^ | 15.1^ab^ | 11.5^b^ | *1.9* | *0.01* | *0.01* | *0.85* |
| C13:0 | 562^a^ | 362^b^ | 377^b^ | 281^b^ | *68.4* | *0.02* | *0.02* | *0.30* |
| C14:0 | 261 | 264 | 250 | 218 | *44.1* | *0.74* | *0.26* | *0.97* |
| C14:1 | 23.6^ab^ | 33.7^a^ | 21.3^b^ | 18.5^b^ | *5.1* | *0.02* | *0.02* | *0.39* |
| C15:0 | 238 | 329 | 380 | 337 | *114* | *0.81* | *0.78* | *0.38* |
| C16:0 | 7854^ab^ | 9253^a^ | 7573^b^ | 5444^c^ | *760* | *<0.01* | *<0.01* | *0.950* |
| C16:1 | 480^b^ | 834^a^ | 664^ab^ | 350^c^ | *118* | *<0.01* | *<0.01* | *0.27* |
| C17:0 | 121 | 123 | 138 | 155 | *26.8* | *0.62* | *0.20* | *0.73* |
| C17:1 | 32.7 | 54.9 | 46.3 | 30.3 | *10.8* | *0.12* | *0.10* | *0.40* |
| C18:0 | 3958^a^ | 4049^a^ | 3603^a^ | 2997^b^ | *286* | *<0.01* | *<0.01* | *0.58* |
| C18:1n10t | 86.7 | 146.7 | 146.0 | 79.0 | *33.9* | *0.13* | *0.12* | *0.16* |
| C18:1n9c | 8385^a^ | 9883^a^ | 7940^a^ | 4850^b^ | *1025* | *<0.01* | *<0.01* | *0.98* |
| C18:1n15t,10c | 946^a^ | 930^a^ | 810^a^ | 655^b^ | *72.9* | *<0.01* | *<0.01* | *0.37* |
| C18:2n6c | 14918^a^ | 17586^a^ | 14160^a^ | 8720^b^ | *1830* | *<0.01* | *<0.01* | *0.97* |
| C20:0 | 63.1^b^ | 99.3^a^ | 65.9^b^ | 61.9^b^ | *13.0* | *0.02* | *0.10* | *0.70* |
| C18:3n6 | 140 | 189 | 108 | 124 | *29.6* | *0.06* | *0.19* | *0.19* |
| C18:3n3 | 1726^a^ | 2098^a^ | 1631^a^ | 733^b^ | *270* | *<0.01* | *<0.01* | *0.85* |
| C20:2 | 355^a^ | 309^a^ | 303^ab^ | 248^b^ | *27.7* | *0.02* | *<0.01* | *0.53* |
| C22:0 | 316 | 371 | 350 | 277 | *44.9* | *0.14* | *0.04* | *0.49* |
| C20:3n6 | 143 | 257 | 149 | 221 | *51.9* | *0.11* | *0.71* | *0.35* |
| C22:1n9 | 143 | 152 | 105 | 128 | *43.6* | *0.73* | *0.72* | *0.35* |
| C23:0 | 2619^a^ | 2632^a^ | 2330^a^ | 1969^b^ | *181* | *<0.01* | *<0.01* | *0.38* |
| C20:4n6 | 117 | 111 | 121 | 135 | *46.6* | *0.95* | *0.57* | *0.96* |
| C24:0 | 372 | 403 | 247 | 285 | *88.9* | *0.22* | *0.26* | *0.12* |
| C20:5n3 | 385 | 342 | 327 | 316 | *125* | *0.97* | *0.73* | *0.78* |
| C24:1n9 | 773^a^ | 829a | 703^ab^ | 559^b^ | *83.5* | *<0.01* | *<0.01* | *0.62* |
| C22:6n3 | 554^a^ | 418b | 405^b^ | 451^b^ | *35.9* | *<0.01* | *0.83* | *0.01* |
| Saturated | 17142^a^ | 17390^a^ | 15752^a^ | 12200^b^ | *1107* | *<0.01* | *<0.01* | *0.78* |
| Unsaturated | 30140^a^ | 34119^a^ | 27883^a^ | 17709^b^ | *3241* | *<0.01* | *<0.01* | *0.93* |
| MUFA | 11424^a^ | 12746^a^ | 10616^a^ | 6760^b^ | *1176* | *<0.01* | *<0.01* | *0.98* |
| PUFA | 18716^a^ | 21373^a^ | 17267^a^ | 10949^b^ | *2130* | *<0.01* | *<0.01* | *0.90* |
| Bacteria | 3786^a^ | 3639^a^ | 3519^a^ | 2807^b^ | *299* | *0.02* | *<0.01* | *0.99* |
| Total | 47282^a^ | 51509^a^ | 43635^a^ | 29909^b^ | *4097* | *<0.01* | *<0.01* | *0.88* |

Dissimilar letters denote statistical differences between groups when overall treatments statistical effect was with P ≤ 0.05.

**Figure S1**. Pasty vent score reference

**Figure S2**. Survival of broiler chickens receiving 0 (CTR), 5 (SHB5), 10 (SHB10), or 20 (SHB20)% dietary spent hemp biomass (SHB).

**Figure S3**. Body weight in male and female broiler chickens receiving 0, 5, 10, or 20% dietary spent hemp biomass (SHB). Letters denote statistical differences (p<0.05) between male and female groups. * denotes P<0.05 between males and females at the same time point when an interaction Sex×Time was significant (p<0.05).

**Figure S4**. Pasty vent score in male and female broiler chickens receiving 0, 5, 10, or 20% dietary spent hemp biomass (SHB).

**Figure S5**. Breast weight, triglyceride content, and quality of the meat in male and female broiler chickens receiving 0, 5, 10, or 20% dietary spent hemp biomass (SHB). * denotes a significant (P<0.05) difference between males and females at a specific SHB dose when the SHB×Sex is with a P<0.05. The p-value reported in the graph is the overall Sex and SHB×Sex effect.

**Figure S6**. Internal organ weights, expressed in weight (g) and percentage of body weight (g/kg BW) of male and female broilers fed diets containing 0, 5, 10, or 20% spent hemp biomass (SHB), respectively. The p-value reported in the graph is the overall Sex and SHB×Sex effect.

**Figure S7**. Transcription of genes related to lipogenesis and lipid oxidation in the internal adipose tissue of male and female broilers fed diets containing 0, 5, 10, or 20% spent hemp biomass (SHB), respectively. *L* denotes a significant (p<0.05) linear effect of the SHB dose. * denotes a significant (P<0.05) difference between males and females at a specific SHB dose when the SHB×Sex is with a P<0.05. The p-value reported in the graph is the overall Sex and SHB×Sex effect.
